# Technology and the Situationist Challenge to Virtue Ethics

**DOI:** 10.1007/s11948-024-00474-4

**Published:** 2024-03-27

**Authors:** Fabio Tollon

**Affiliations:** 1 Department of Philosophy, University of Edinburgh, Edinburgh, UK; 2 Department of Philosophy, Unit for the Ethics of Technology, Center for Applied Ethics, Stellenbosch University, Stellenbosch, South Africa; 3 Centre for Artificial Intelligence Research (CAIR), University of Pretoria, Pretoria, South Africa

**Keywords:** Situationist challenge, Emerging technology, well-being

## Abstract

In this paper, I introduce a “promises and perils” framework for understanding the “soft” impacts of emerging technology, and argue for a eudaimonic conception of well-being. This eudaimonic conception of well-being, however, presupposes that we have something like stable character traits. I therefore defend this view from the “situationist challenge” and show that instead of viewing this challenge as a threat to well-being, we can incorporate it into how we think about living well with technology. Human beings are susceptible to situational influences and are often unaware of the ways that their social and technological environment influence not only their ability to do well, but even their ability to *know* whether they are doing well. Any theory that attempts to describe what it means for us to be doing well, then, needs to take these contextual features into account and bake them into a theory of human flourishing. By paying careful attention to these contextual factors, we can design systems that promote human flourishing.

## Emerging Technology and AI

It is often unclear what the ‘effects’ of a given emerging technology might be. Part of the reason for this is that such technologies are still in the nascent stages of their development, and we simply do not know exactly how they will come to feature in our lives and societies. This, of course, does not mean that we give up. We find sophisticated methods and approaches in the literature that illustrate how to deal with this inherent ‘uncertainty’ that comes with emerging technology (Schot & Rip, 1997; Grunwald, 2011; Guston & Sarewitz, 2002). I aim to make a philosophical contribution to this debate by providing a framework for appreciating the role that technological design plays in the cultivation of virtue. Virtue, however, is not easy and, as many suggest, is easily undermined.

In this paper I therefore outline some implications of the so-called ‘situationist challenge’ to virtue ethics in light of emerging technologies, such as AI. To do this, I first outline some general characteristics of emerging technology. Second, I describe the ‘soft’ impacts of technology, and how these impacts contribute to further uncertainly regarding technological assessment. Third, I suggest one way of dealing with these soft impacts: adopting a eudaimonic conception of well-being. Fourth, I expand on this notion of well-being and defend it from a common criticism in the literature, the so-called ‘situationist challenge’. Last, then, I apply my discussion to self-tracking technologies.

My analysis here will apply to emerging technologies more generally. Therefore, I will first outline some key characteristics of emerging technologies, and describe what makes them especially difficult to evaluate. Of course, it is very difficult to make any general claims about ‘technology’, as each technology will have its own unique characteristics and effects. However, it is nonetheless important that I provide some general support to the idea that *emerging* technologies are especially deserving of our attention. After this I will turn to the question of what it might mean to say we are living well with them, using the specific example of social media technologies and the notion of “digital well-being” (Burr & Floridi, 2020; Burr et al., 2020Dennis, 2021a, b; Steinert & Dennis, 2022). This approach is in line with many developments in the philosophy of technology, where researchers are becoming increasingly concerned with understanding the relationship between technology, values, and the way(s) in which we live (Vallor, 2016; Klenk & Hancock, 2019; Klenk, 2020; Johnson, 2022; Steinert & Dennis, 2022).

### Promises and Perils

Acknowledging the diverse factors that come to influence technologies is the first step to understanding the complexity of *emerging* technologies. These are nascent technologies whose meaning, use, and impact is still *unstable*, where we do not yet know what their *future* impacts might be. For example, take the emergence of humanoid robots. These are robots, often equipped with advanced AI, whose future use and impact remains relatively undecided. Will they be used as “sex robots” and compete with human sex workers? Will they replace human care workers, and what might the impact of this be on patients and the job market? Is it even ethical to design and produce humanoid robots in light of the “attribution bias” displayed by human beings? (Levy, 2007; Danaher, 2016; Coeckelbergh, 2021; Müller, 2021). The answers to these questions cannot be settled easily. More importantly, however, how we answer these questions will reflect what we imagine the future to be like (Tollon, 2022).

A common feature of such speculation, however, is that emerging technologies are often framed in terms of their *promises* or *perils* (Johnson, 2022). The predictions of what a technology may afford are grounded in what they promise us (better health, more ecologically friendly living, a world without work) or their perils (reduced autonomy, AI overlords, threats to democracy). This is due to a few features particular to emerging technologies. First, these technologies are in the early stages of their development, and so their current state of development and use are not necessarily indicative of their future potential (although this will of course inform our speculation). The current status of the technology is therefore only an indication as to what its final stable form might be. Thus, people who are in favour of the technology (or those who are against it) can use this to their advantage by speculating (and perhaps embellishing) their accounts of what the impact of the technology might be. Morality and technology, therefore, couple together in a way that complicates our ability to clearly distinguish between the two (Boenink et al., 2010).

Moreover, technologies can come to “mediate” morality (Verbeek, 2006), which can lead to “technomoral change”, in which “technology and morality *mutually* shape each other” (Swierstra, 2015). This more nuanced understanding of the relationship between morality and technological development permits us to identify and potentially address these soft impacts. Moreover, the phenomenon of technomoral change goes hand-in-hand with what Shannon Vallor refers to as “technosocial opacity” (Vallor, 2016). This can be understood as a kind of blindness brought on by the complex interaction of new and old technologies, and how these come to bear on our institutional and cultural practices. This technosocial ‘blindness’ has been accelerated recently by the *convergence* of various advances in AI and related fields, which follow from

discrete technologies merging synergistically in ways that greatly magnify their scope and power to alter lives and institutions, while also amplifying the complexity and unpredictability of technosocial change (Vallor, 2016).

It is for these reasons that trying to answer the question of whether we are living well with technology, and especially emerging technology, is no simple matter.

## The ’Soft’ Impacts of Technology

Due to the two essential features noted above regarding emerging technologies, it is not possible to simply look at the ‘hard’ impacts that these technologies might have. ‘Hard’ impacts here refer to impacts that can answer to three conditions: quantifiability, clear and noncontroversial harm, and direct causation (Swierstra, 2015). For example, if a private company is polluting a nearby river, it would be possible to take water samples from the river, show that the chemicals from the company’s factory are in the river, and make a causal link between this and detrimental effects that might be observed in the river (loss of biodiversity, pollution of drinking water, increase in diseases associated with the chemicals in question, etc.). Thus, the effects can be quantified, there is a clear harm, and causation can be established. If the impacts of a technology admit to such a description, we can therefore define its effects as sufficiently ‘hard’, and this allows us not only to implement relatively standard mechanisms of responsibility and the allocation of accountability, but also to assess how it might affect human well-being. These ‘hard’ impacts, and their evaluation, are best suited for “stable” or “entrenched” technology. These are technologies whose use, meaning, and place in society is relatively well-established. For example, we know that CO_2_ emissions from large-scale animal agriculture are very damaging for the environment, notwithstanding the increases in food production we have seen over the years. However, such a ‘balanced’ perspective usually emerges after some time has passed and the technology has become ‘stable’, in the sense that its uses and effects are relatively well understood. We now understand, better than we did in the 1920s, for example, the disastrous effects of fossil fuels and CO_2_ emissions. We can see that the technology at some point provided a benefit, but that the costs now outweigh those benefits. For emerging technologies, however, such a ‘cost-benefit’ approach might not be possible in practice, due to the ‘soft’ impacts of technology. Soft impacts, by their very nature, are difficult to quantify, do not have clear and noncontroversial harms, and their causal impact is difficult to trace. For example, it has become common to attribute the seeming rise in political polarization to new forms of social media and their governing algorithms (such as Twitter, Facebook, Instagram etc.). However, it is not so easy to get a handle on the way social media platforms influence levels of polarization. Alberto Acerbi, for example, argues that we have been seeing a rise in political polarization that extends to well before the introduction of social media, and that offline individuals might in fact be *more* polarized than those that spend their time online (Acerbi, 2020).

In this example, therefore, we see that the ‘impact’ of the technology is difficult to quantify, as it is still an open question as to what the exact effects of social media might be on polarization. Second, and following from the aforementioned, it is unclear what exactly the harm might be, especially if it is the case that social media use in fact *decreases* polarization. And third, establishing direct causation in such a situation seems especially complicated, if not impossible. This is not to say that the issue is settled: with or without social media, measuring levels of polarization is difficult in itself as, for example, should it be done from a group level or the level of the individual. Moreover, the study of social media is still a recent undertaking for researchers. It is important to note, however, that the problem of causality might be a more general problem. Even if we have a stable technology, it might still be difficult to establish the causal relations that lead to some outcome (such as polarization), and it seems this would be true in most cases when we try to explain and understand social phenomena. The point, however, is that these ‘soft’ impacts can make this situation worse.

Soft impacts, such as the intersection of social media and polarization or ‘echo chambers’ are thus substantially more difficult to get a handle on than hard impacts. Other, easy examples might be how Facebook influences friendship, or whether the internet will negatively impact certain intellectual virtues. In all of these cases what we observe is an inherent ambiguity and *contestability*, and, perhaps most importantly, part of the effects we are attempting to measure are *produced by the agents themselves*. Thus, unlike in the case of a factory polluting a river, when it comes to soft impacts, users more intimately co-produce the harms (or benefits) that are the object of our study (Swierstra, 2015).

One might wonder, however, what the metaphysical relationship is between hard and soft impacts. More precisely: is this a difference in kind, or only a matter of degree? For the purposes of this argument, I will assume that the distinction between ‘soft’ and ‘hard’ impacts can be understood as representing two sides of a sliding scale. Some technologies might be on the ‘soft’ side while others are on the ‘hard’ side, and the difference between them is a matter of degree. Moreover, at least in the case of many emerging technologies, soft impacts are something that we have to live with. Over time, however, these impacts might become ‘harder’, as we gain a greater understanding of how the technology affects us. However, this does not necessarily mean that hard impacts are easier to deal with. Even if the impacts of these technologies are more predictable, it might still be incredibly difficult to address the problems they raise (for example, the technologies that run on fossil fuels and exacerbate climate change). Nevertheless, what I propose here is rather modest: I do not claim that that my framework can turn soft impacts into hard ones. Rather, my hope is that the framework can help us live with these soft impacts.

## What to do about Soft Impacts

Now that we have a general idea of what soft impacts involve and why they are important, we can attempt to chart a course through the various effects such technologies might have. As noted above, because these effects are *co-produced* by human users, it behooves us to make these users central to our investigation. One way to get a handle on these effects might be to investigate the effects that these technologies have on users’ well-being. This is quite a common strategy, and those who study the effects of technology often use metrics related to feelings of self-esteem, such as whether agents are ‘doing well’, or how technology might influence their ‘social capital’ (Wellman et al., 2001; Valkenburg et al., 2006). To measure these variables, however, it is important that we grasp what it means for agents to be ‘doing well’ in the first place, and there are better and worse ways of conceiving of well-being. Perhaps the most intuitive way of thinking about ‘well-being’ is in terms of happiness, and perhaps the most intuitive account of happiness is simply to have numerous pleasurable experiences (Frijda, 1986; Mulligan & Scherer, 2012). Such a ‘hedonic’ conception of well-being, however, has some serious issues.

I will argue that ‘hedonic’ accounts of well-being are flawed, and that we should instead adopt a ‘eudaimonic’ conception of well-being (Kahneman et al., 1999; Steinert & Dennis, 2022). I do this by first fleshing out what hedonic well-being might entail and showing how it is different from eudaimonic conceptions of well-being.

### Hedonic and Eudaimonic Well-Being

The first issue to get a handle on is what exactly we might mean for an agent to be ‘doing well’. Hedonic approaches have a very thin understanding of what it means to be ‘doing well’ or what constitutes ‘well-being’, and often conceive of it as whether agents have, on average, more pleasurable rather than painful feelings when interacting with technology (Kahneman et al., 1999). This is a natural and intuitive way of trying to measure well-being, and while the rise in empirical reflection on the effects of technology is certainly welcoming, such a narrow, hedonistic, focus is problematic. Life satisfaction surveys that are used to produce ‘robust’ constructs such as “psychosocial well-being” have a utilitarian focus and thus assume an impoverished account of what it might mean to be ‘doing well’ or ‘flourishing’ (Vallor, 2010).

On such hedonistic accounts, it is argued that we should understand well-being “in terms of the attainment of pleasure and the avoidance of pain” (Steinert & Dennis, 2022). So, a life ‘lived well’ in this sense is one that has more positive than it does negative emotions. This seems intuitive enough, as it does seem we can sort our emotions into those that are pleasurable and those that we would rather avoid. Often, this focus is justified because of the “hedonic tone” or “valence” of emotions more generally (Frijda, 1986; Mulligan & Scherer, 2012).

While I think this understanding of emotions is rather crude and unsatisfactory, a more general problem is this: the hedonic approach seems to ignore the fact that we care about more than just pursuing pleasure and avoiding pain. This is of course not a new point. However, the deeper concern is that hedonic accounts do not present us with a satisfying presentation of our emotions in the first place (as compared to eudaimonic accounts). As Steinert and Dennis argue, “concentrating on eudaimonic well-being has advantage because eudaimonic theories have a much broader conception of the role that emotions play in well-being, viewing a good life as one that is open to positive and negative emotions” (2022).

A concrete example of this, as it relates to emerging technology specifically, comes from social media. The design of social media technologies has an overwhelmingly hedonic focus, as the goal of many of these platforms is to facilitate the experience of short-term pleasure by users. Separate from the design of these platforms, users themselves also make use of social media for their own hedonic reasons: sharing personal achievements and news, and having this seen and acknowledged by others, can induce positive emotions such as pride (Steinert & Dennis, 2022). The point is that it is easy (and perhaps even intuitive) to attempt to understand social media technologies in a hedonistic way. However, such an approach has serious shortcomings.

For example, sometimes the pursuit of what we find valuable and meaningful in our lives can be painful. As an example, consider that I aim to run a marathon. To achieve this goal requires many sacrifices. I might have to wake up early on most days to go running, my social life might suffer because I am too tired to see my friends in the evenings, and on weekends when I could be sleeping, I have to head out the door and spend hours putting one foot in front of the other. Of course, there will be moments during this process that I feel good and proud of myself for what I am doing. After a particularly good session I might even think that the training is worth the sacrifices I am making. However, the fact remains that there are many unpleasant experiences involved in marathon training. Yet, many people pursue such goals despite these negative experiences. The same could be said of those who do bodybuilding or make demanding career choices. Conversely, there are many things that are immensely pleasurable that have deleterious effects on well-being, such as some hard drugs. Hedonic approaches, therefore, have a restrictive and ultimately implausible account of what it means to be ‘doing well’. This is especially problematic when we attempt to tease out the potential effects that technologies can have on our ability to live well and flourish. What we really want to know about these systems is whether they enable users to ‘do well’ in a morally important sense (Vallor, 2010).

Users might indeed truthfully report that frequent usage of, for example, social media, increases their well-being by allowing them to stay in contact with friends who live far away, or that they receive emotional support from other users on these platforms. However, it might also be the case that for these users certain virtues are repressed (such as self-reliance) or they may even develop certain vices (such as arrogance) due to their use of the technology (Vallor, 2010). While Facebook might allow friends who now live far away from each other to stay in contact, it might also lead those same users to be less open to new experiences. The point is not to go into specific details here, but rather to see that merely measuring various psychosocial metrics is not sufficient for us to get a grip as to what might be happening to the person’s *character* as they interact with technology, and nor can we expect this process to be linear, deterministic, or the same for all users. Persons with diverse cultural backgrounds or natural dispositions may have different ways of interacting with technology, and we ought to be mindful of this.

Simply understanding how technologies might affect agents’ mental states or psychological well-being, while important, does not give us a full handle on how their moral development may be impacted for example, by their *continued* use of the technology. That is, we lose nuance by leaving out how technologies enable certain kinds of behaviours, and, more importantly, how these behaviours can become *habits*. The importance of habits for the ‘good life’ is well known and is especially prevalent in virtue ethical accounts of morality. For example, Aristotle, when describing the virtues, claimed that they are “made perfect by habit” (Aristotle, 2009). Therefore, morally virtuous action is not about mere rule following, nor is it only about what an agent does, but rather how an agent performs an action (if, for example it is done voluntarily, deliberately, or with joy). The focus for Aristotle is the *temporal* cultivation of right action (and right thought), with the goal that virtuous states come start to come about. Therefore, once-off measures are not sufficient to capture the impacts that technologies may have on our ability to live well. We require both empirical studies that attempt to track the habits that certain technologies might induce, and philosophical reflections on the ways in which technologies could potentially impact our habits and the expression of virtues over time. This prompts us to reflect on the effects that technology might have on our *dispositions*, not just when interacting with technology, but in our other engagements. Shannon Vallor puts it well when she suggests that

by narrowly focusing on tallies of losses and gains in social capital and other measures of psycho-social well-being, it is easy to overlook what is happening to the persons purportedly enjoying these gains and suffering these losses, that is, what is happening to their character. (2010: p. 163)

A good example of such an approach was taken in a recent study conducted in the United Kingdom (UK) under the auspices of the University of Birmingham. The goal of the study was not to measure users’ levels of satisfaction, but rather to try and uncover what the effects of social media use may be on the moral character of children, with a particular focus on empathy and honesty (Morgan & Kristjánsson, 2017), and how different parental styles can come to promote morally virtuous behaviour in children.

Another reason to move beyond simple hedonic conceptions of well-being is the fact that they cannot capture that essential feature of human life: meaning. It is undeniable that some things *matter* to us more than others, and that this cannot be neatly explained by the self-interested presuppositions of hedonic conceptions of well-being (Wolf, 2010). My point, however, is more basic. Some things, some relationships, some books, simply matter more to us than others, and this mattering might of course be influenced by the positive states of mind that these things produce. However, such a simplistic reading would not capture what they mean to us. For example, drinking a coffee in the morning might produce the same amount of pleasure as giving advice to a friend, but it seems clear that the latter is more meaningful than the former. Moreover, this can even hold in cases where there is a large asymmetry in the hedonic profiles of the activities. For example, binging the latest product of popular culture might be a pleasurable experience that could last many hours. Compare this with participating in a democratic election, which might involve many tedious activities, such as queueing for many hours or registering to vote. The one might have a better hedonic profile than the other, but I would hope that participation in democracy should matter more. What these examples suggest (and of course you may not agree with all of them) is the basic idea that the virtuous life is the better life to lead, all things considered. This is true even in those cases where we have to say no to certain pleasures in specific contexts.

All this suggests that we would be better off adopting *eudaimonic* conceptions of well-being. Adopting such a perspective allows us to account for and explain why many things that we find meaningful in life (and that we find are worth pursuing) can cause us discomfort. In fact, eudaimonic approaches explicitly acknowledge that negative experiences are necessary for a meaningful life (Steinert & Dennis, 2022). Such eudaimonic perspectives naturally draw on the Aristotelean notion of *eudaimonia*, which is often translated from Greek as ‘happiness’ in English versions of Aristotle’s work. It is accepted that his term encompasses far more than our modern, mostly psychological, concept of happiness does. Eudaimonic well-being is therefore not merely hedonic or simply about pleasurable mental states. Rather, here we are concerned with a kind of *flourishing*, which incorporates the degree to which we can be said to be virtuous or whether or not we have achieved our life’s goals (whatever these might be, and assuming that they are morally appropriate). For Aristotle, the virtuous person, when flourishing, is objectively *happy* (Aristotle, 2009). Thus, *eudaimonic* perspectives broaden the notion of well-being so that it might include aspects such as meaningfulness and account for why some valuable goals might require us to go through painful experiences.

To go back to the example of marathon training, a eudaimonic perspective explains why the discomfort that comes with training for a marathon can indeed be part of a meaningful life and promote well-being. The habitual training, which might involve short term discomfort (such as waking up early, muscle soreness, and social sacrifices) nonetheless lead to the attainment of meaningful life goals, such as the successful completion of a marathon. Significantly, it is not just the attainment of the goal that makes this pursuit meaningful: it is precisely in the cultivation of good habits over time that we might start to see changes in our character. In order to do this, we need to have a basic *understanding* of what our values and commitments are, and put into place strategies for realizing them, even if we know that parts of these strategies will involve discomfort. To flourish, then, is not simply to be chasing pleasure all the time.

However, one might object that this eudaimonic account falls prey to a similar critique that I presented against hedonic accounts. For hedonic accounts, I argued that because ‘pleasure’ and ‘pain’ are not given directly, they cannot enter into a calculation here. However, we might be able to level the same critique at eudaimonic perspectives: how are phenomena like ‘value’ and ‘meaning’ presented to us? They, too, seem not to be directly given. Thankfully, the eudaemonist has a response: practical wisdom, or judgement, is a plausible candidate to fill this gap. To see how this is the case, we can call on one of the most popular defenders of virtue ethics, Aristotle.

For Aristotle, the properly virtuous person is capable of successfully moderating their responses to differing situations in an intelligent and thoughtful manner, and the virtuous skill required for this he termed *phronesis* (Aristotle, 2009). *Phronesis* has been translated in a number of ways, such as prudential reason, prudence, or practical wisdom (Vallor, 2016). It essentially boils down to the ability to properly discern the morally relevant contours of a given situation and strike a *balance* in one’s response to a given practical situation. That is, one should be able to respond without excess or deficiency, and the virtuous person is characterized by their ability to strike the appropriate mean in their dealings with others. For example, someone who has “good temper” strikes a mean with respect to anger (Aristotle, 2009). They respond with anger to the ‘right things’ (for example, injustice), but not to other things (for example, something trivial like the colour of another person’s shirt). Such appropriate responses are to be praised, and the inverse would naturally be the cause of blame. As Aristotle puts it,

the good-tempered man tends to be unperturbed and not to be led by passion, but to be angry in the manner, at the things, and for the length of time, that reason dictates; but he is thought to err rather in the direction of deficiency; for the good-tempered man is not revengeful, but rather tends to make allowances. (Aristotle, 2009: p. IV. 5)

The good-tempered person, therefore, is capable of discerning when it is appropriate to be angry, and this reveals the centrality of some form of *reasoning* to virtue ethics. We might characterize this reasoning as a form of *judgement*, whereby agents are capable of developing the skills and habits required of them to properly apprehend how to live a valuable and meaningful life. The virtuous agent, however, has to get it right on a number of scales: they also need to have a handle on how proportionate a response ought to be, against whom a respond might be required, etc.

The basic takeaway here is that if we want to get a proper handle on what it means to “live well” or increase “well-being” we cannot simply aim to maximise the number of pleasurable experiences that we have. This of course has implications for how we go about assessing the effects of technology. It means that even if technologies such as social media make users “feel good” this does not tell us, necessarily, to what extent it might influence their overall well-being. Conversely, just because people might report *negative* feelings associated with social media does not tell us, either, whether it might be harmful. This complicates things, but, as I have argued above, a eudaimonic perspective gives us some resources for dealing with these issues. A problem with such eudaimonic perspectives, however, is that they would assume that there are reliable dispositions at the heart of our decision making. That is, for us to flourish we ought to act virtuously, but this of course presupposes that we *can* act with virtue. This might be undermined by the fact that human beings are heavily susceptible to environmental influences in their decision making.

## Situational Influences

The designers of certain technologies *actively* try to influence users into behaving in certain ways. This might happen even in innocuous cases because it is simply in the interests of the producers of technology to maximise the revenue that their products can generate and being able to reliably predict and direct the actions of users is an effective way to achieve this goal. Therefore, even without malicious intent on the part of designers, we still might find ourselves as users in a position where we are being herded to behave in certain ways. This is not because we are ignorant in a sense in which we are blameworthy, but rather that our ignorance is utterly predictable, and, indeed, often a *feature* of our engagement with technologies such as social media platforms.

Operating systems on smartphones or laptops are illustrative of this, as these are technologies that are *designed* in specific ways to keep users *engaged* with them. For example, on Apple’s “Human Interface Guidelines”, they suggest that designers “find the correct balance between enabling users and avoiding unwanted outcomes. An app can make people feel like they are in control by keeping interactive elements familiar and predictable”, and that “it’s *usually* a mistake for the app to take over the decision-making” (emphasis mine)^1^. This kind of design imperative is informed by empirical studies which maintain that there are various cues that can be used in order to facilitate users having the experience of agency. These cues are *predictability* and *fluency* (Madary, 2022). Naturally, if a system is predictable, users will feel as though they are in control of what is happening (even if they are not). Similarly, if a system’s design is fluent then users might experience it as an extension of themselves rather than as a discrete device with its own properties.

This makes sense, as from a creator’s perspective, keeping users engaged and using their platform is in their best interests. This is short of making the claim that these online systems can be used to *manipulate* us, but it is hard to deny that they can have a degree of influence on us (Klenk & Hancock, 2019; Susser et al., 2019). An easy example of this kind of influence might be the ways that online advertisers can collect and aggregate large amounts of user data in order to generate better targeted adverts so that users are influenced in the most effective way possible (Susser et al., 2019). This kind of challenge is important in the context of emerging technologies, especially in online environments. Consider the myriad situational influences that users of the internet are exposed to everyday: targeted adverts, filtered newsfeeds, and information bubbles all contribute to creating potentially unfavourable epistemic environments for agents to properly and reliably exercise any virtuous dispositions that they might have. For example, a recent study on Twitter found “a remarkably consistent trend: In six out of seven countries studied, the mainstream political right enjoys higher algorithmic amplification than the mainstream political left” (Huszár et al., 2022). If right-wing content is amplified on Twitter to a larger degree than other political content, this makes it more difficult for users to be exposed to opposing points of view and might encourage confirmation bias. This would be especially troubling if humans are more easily influenced by the situations that they find themselves in rather than the stable dispositions they might have. For example, traits such as generosity, arrogance, and bravery are dispositions to react in particular ways to certain trait-eliciting circumstances, but if features of the environment can better explain and predict such behaviours, then the significance of human character could be questioned (Alfano, 2013b).

This is important for my purposes because the idea of eudaimonic well-being, and the account of what constitutes flourishing that follows from it, rides on the fact that there are certain dispositions that are praiseworthy and worth inculcating in agents. If our external context and environment determine our behaviour, then appeal to internal features such as character or disposition is superfluous. Significantly, this would also mean that our commitments and values are not really “ours” in the sense required above, and so attempts to get a handle on what it would mean to flourish would indeed be superfluous. Unfortunately, there is a large body of evidence that situational influences trump dispositional ones: let us call this the “situationist challenge” (Harman, 1999; Doris, 2002; Alfano, 2013b). According to this challenge, the context in which a behaviour occurs can better explain the behaviour than appealing to any dispositional features of the agent. This is a problem for getting a handle on what it means to live well with technology as it could mean that no matter how “virtuous” we might be, situations determine our behaviour.

A way to respond to this kind of challenge might be to insist that the evidence from social psychology (and perhaps even behavioural economics) that suggests the inadequacy of our dispositions to predict and explain our behaviour is unconvincing (Kahneman, 2011). There are a number of ways that this defence could be articulated. One could claim that the evidence constitutes a category mistake, and that the existence of character traits should be informed by *introspection* instead of scientific measurement (Annas, 2003). Or one could claim that the standard experiments that purport to show that we are at base not virtuous do not track morally important behaviour, or that once-off experiments, such as those in social psychology studies, do not track what we mean by virtue, which would require longer term studies.

Another option, however, is to accept the challenge and attempt to integrate it into a broader theory of human flourishing. That is, instead of trying to explain away the impressive array of evidence, we might instead try and use it to our advantage. While this does not necessarily get us a full theory of what exactly the virtues are, or how precisely to cultivate them, it gets us closer to *sustaining virtuous behaviour*.^2^ I think that this is a very promising strategy, and that attempting to flesh out such a theory yields interesting results, especially as these concern important relations between human agents, their environment, and the social milieu in which they find themselves (Alfano, 2013a).

## From Consumers to Producers

Rather than thinking of ourselves as simply the *products* of situational influences, what if we instead saw that we play a significant role in *producing* these situations in the first place? This is the approach suggested by Mark Alfano (2013b) in response to the situationist challenge. This requires a shift from thinking of agents such as ourselves as *consumers* of situations but rather as *producers* of situations (Alfano, 2013b). This shift in perspective is especially fruitful when combined with a sustained reflection on the impacts of technology. Technologies are designed by humans, which means that we are not merely at the whim of our technologies, but can execute a degree of control over how they influence our lives. Thus, we can invoke this new perspective and see how virtue could feature in it.

[r]ather than simply seeking and avoiding situations based on their virtue-conducive properties, we may take a more active role and create (both for ourselves and for others) situations with an eye to their virtue-conduciveness. (Alfano, 2013b)

In this way, we can come to see that “the power of situational influences becomes a tool rather than a threat” (Alfano, 2013b). This insight, therefore, ought to be baked into not only the design but also our use of technological artifacts. For example, we might expect users of social bots to treat these systems with dignity and respect which might enable them to work on sustaining virtuous behaviour.^3^

Significantly, this kind of argument suggests that we look beyond only the agents involved in a situation in order to fully appreciate how we ought to understand human flourishing and how we might sustain virtuous behaviour. For humans to be ‘doing well’ in any meaningful sense we cannot merely analyse the human agents themselves. As I have shown, humans are susceptible to situational influences and are often not aware of the ways that their social and technological environment influence not only their ability to do well, but even their ability to *know* whether they are doing well. Any theory that attempts to describe what it means for us to be doing well, then, needs to take these contextual features into account and bake them into a theory of human flourishing.

## Self-Tracking Technologies

Before concluding it would be useful to consider an example of where this new analytic framework might be applied, namely, self-tracking technologies. These are devices that allow users to keep track of and quantify various aspects of their lives. Often, these devices centre around metrics related to health (in some broad sense). For example, fitness trackers (such as Fitbits) allow users to track their step counts each day, monitor their heart rate, and get an indication of their calories burned (never mind that this latter metric is often unreliable). The idea behind these devices is that they can aid us in living happier, healthier, and more purposeful lives. By helping us keep track of these metrics, the hope is that we would be more motivated to eat healthier food, be more active, and engage better habits, thus contributing to us living more meaningful lives. However, as noted by Tamar Sharon, the debate over the effects of self-tracking on our health (broadly construed) remains largely polarized. It would be far beyond the scope of my paper to canvass this literature here, and, in any case, Sharon does a wonderful job of this in her article. Her point is to make explicit the contested nature of self-tracking technologies. On the one hand, we have the promise of personalised healthcare and its supposed benefits (cheaper, more efficient, etc.). On the other hand, we have the potential ethical, political and social implications of the widespread use of such technologies (Nafus & Sherman, 2014; Vallor, 2016; Sharon, 2017). I believe that the eudaimonic account presented here can illuminate the ethical contours of self-tracking technologies in a number of ways. I think are three significant questions that the eudaimonic perspective that I advocate for suggests we ask.

First, consider the emphasis that this account places on *habit*. This notion of habit is not to be confused with repeated action. Rather, we can understand habit in a broader, Deweyian sense (Dewey, 1957). On Dewey’s account, habits are ways of responding to the world, and of course these often arise through repeated activity (whether this be an action or a thought), but they also structure our *interaction* with the world (1957: p. 25). For Dewey, “the essence of habit is an acquired predisposition to *ways* or modes of response, not to particular acts” (Dewey, 1957). Therefore, with respect to self-tracking technologies, we can ask the question of whether they influence our modes of response in ethically desirable ways. That is, whether they inculcate habits (in this broad sense) that promote flourishing.

Second, and perhaps relatedly, we can shift our perspective from seeing the users of self-tracking technologies as passive consumers to active producers. That is, instead of viewing users as ‘merely’ consuming the data they are presented with, we can see how using these technologies in in fact are being used to *produce* virtue-enabling environments. If people use the technology in the service of better health, then it would be a mistake to think that they are somehow ‘outsourcing’ their health decisions to technology. Rather, the technology, by being used in a thoughtful way, is part of their being virtuous.

Third, the eudaimonic perspective suggests that we ask what the effects of these technologies might be on users’ *judgement* or practical wisdom. Can the data from self-tracking devices contribute to the examined life, and do they help users make better decisions? This question is rather tricky: Shannon Vallor, for example, argues that such devices do not contribute to human flourishing, in the sense that they do not make a genuine contribution to self-knowledge (2016). Vallor contends that certain self-tracking systems distract users from meaningful aspects of their lives by overburdening them with useless information, do not promote an appropriate moral ideal of the self, and only capture easily quantifiable aspects of users lives (Vallor, 2016). While the aforementioned is certainly plausible, it seems to ignore the fact that these technologies might be vehicles in the attainment of virtuous dispositions.

While Vallor is surely right to note that self-tracking *by itself* is insufficient for the inculcation of real virtue, this is not to say that it cannot be used as a means for *achieving* virtue (Wieczorek, 2023). Simply collecting data is of course insufficient for leading an examined life, it is nonetheless possible to use this data in order to examine one’s life. This suggests that the technomoral virtues should leave open the possibility that various technologies might in fact aid us in our decision-making, and thus contribute to practical wisdom or judgement. And it is precisely this kind of insight that the account I have developed in this paper encourages. By reconfiguring ourselves as producers *and* consumers of our environment, we come to see technology as an important part of human relations. While human agency is certainly an important part of these relations, it is not the whole story. Not only can technology have certain effects on users, but users may use technology as a tool to achieve a variety positive outcomes, such as tracking how much time they spend with family vs. strangers (Nafus & Sherman, 2014; Sharon, 2017; Wieczorek, 2023). This illustrates how technological ubiquity might not be *necessarily* alienating.

## Conclusion

In this paper I have argued that getting a handle on whether we are ‘living well’ with emerging technology is no easy task. This is due, in part, to the ‘soft’ impacts of these technologies. However, it is nonetheless possible to make a start in this process by attempting to articulate these soft impacts and framing them in terms of their promises and perils. However, this framework does not settle the issue of what it means to be living well with technology: to do this we must have an idea of what “well-being” means. Here I argued for a eudaimonic conception of well-being. This conception, however, is built on the idea that we have stable character traits. This theory is therefore vulnerable to the “situationist challenge”. In response to this challenge, I suggested that instead of viewing it as a threat we incorporate it into our thinking about how we live with technology. This reveals how human agency is not just in our heads but is bound up in contextual and situational features. These features not only structure the possibilities for action in each situation but are intrinsically part of human agency.

## Data Availability

Not applicable. No datasets were generated during and/or analysed in the preparation of this publication.

## Footnotes

1 This information can be found at https://developer.apple.com/design/human-interface-guidelines/ios/overview/themes/, accessed 13 April 2022.

2 Thanks to Ivan Bock for this point.

3 I am thankful to an anonymous reviewer of this journal for this example.
